# Suppression of Mic60 compromises mitochondrial transcription and oxidative phosphorylation

**DOI:** 10.1038/srep07990

**Published:** 2015-01-23

**Authors:** Rui-Feng Yang, Li-Hong Sun, Ran Zhang, Yuan Zhang, Yu-Xuan Luo, Wei Zheng, Zhu-Qin Zhang, Hou-Zao Chen, De-Pei Liu

**Affiliations:** 1 State Key Laboratory of Medical Molecular Biology, Department of Biochemistry and Molecular Biology, Institute of Basic Medical Sciences, Chinese Academy of Medical Sciences & Peking Union Medical College, Beijing 100005, P.R. China

## Abstract

Precise regulation of mtDNA transcription and oxidative phosphorylation (OXPHOS) is crucial for human health. As a component of mitochondrial contact site and cristae organizing system (MICOS), Mic60 plays a central role in mitochondrial morphology. However, it remains unclear whether Mic60 affects mitochondrial transcription. Here, we report that Mic60 interacts with mitochondrial transcription factors TFAM and TFB2M. Furthermore, we found that Mic60 knockdown compromises mitochondrial transcription and OXPHOS activities. Importantly, Mic60 deficiency decreased TFAM binding and mitochondrial RNA polymerase (POLRMT) recruitment to the mtDNA promoters. In addition, through mtDNA immunoprecipitation (mIP)-chromatin conformation capture (3C) assays, we found that Mic60 interacted with mtDNA and was involved in the architecture of mtDNA D-loop region. Taken together, our findings reveal a previously unrecognized important role of Mic60 in mtDNA transcription.

Mitochondria have their own genome comprised of mitochondrial DNA (mtDNA), which encodes 13 essential proteins within the oxidative phosphorylation (OXPHOS) complexes in vertebrates^1^,^2^,^3^,^4^. The transcription of these proteins relies on the basal mitochondrial transcription machinery, which consists of mitochondrial transcription factor A (TFAM), mitochondrial transcription factor B2 (TFB2M) and mitochondrial RNA polymerase (POLRMT)^5^,^6^. Human mtDNA has only one promoter region within the non-coding D-loop region. To initiate mtDNA transcription, TFAM binds to the promoter region and induces a dramatic U-turn in the mtDNA, which helps to form a specific higher-order conformation in the D-loop region and places the C-terminal tail of TFAM next to the transcription start site^7^,^8^. Then with the combination of TFB2M and POLRMT, TFAM initiates mtDNA transcription^6^,^9^,^10^. Precisely regulated mtDNA transcription is required for OXPHOS. Deregulation of mtDNA transcription causes various diseases and aging due to a severe impairment of respiratory function^11^,^12^. Hence, a deeper understanding of mtDNA transcription is of great importance for human health.

Mic60, also known as Mitofilin, HMP or Fcj1, is a mitochondrial inner membrane protein first identified in the heart^13^. As a crucial component of the mitochondrial contact site and cristae organizing system (MICOS), Mic60 has been well characterized in controlling mitochondrial morphology^14^,^15^,^16^. Mic60 plays important roles in many aspects of mitochondrial functions. Suppression of Mic60 increases mitochondrial membrane potential and the production of reactive oxidative species (ROS)^14^. We previously reported that Mic60 also regulates cytochrome c release during apoptosis^17^. Recently, we found that Mic60 is involved in the development of cardiomyopathy, and that Mic60 overexpression promotes cardiac hypertrophy in response to hypertrophic stimuli^18^. However, the physiological behavior of Mic60 and the mechanism how Mic60 functions remain incompletely understood.

In the present study, we report that Mic60 interacts with mitochondrial transcription factors and Mic60 deficiency decreases TFAM binding to mtDNA promoters. In this manner, suppression of Mic60 compromises mitochondrial transcription and OXPHOS activities.

## Results

### Mic60 interacts with mitochondrial transcription factors

**Figure 1:**
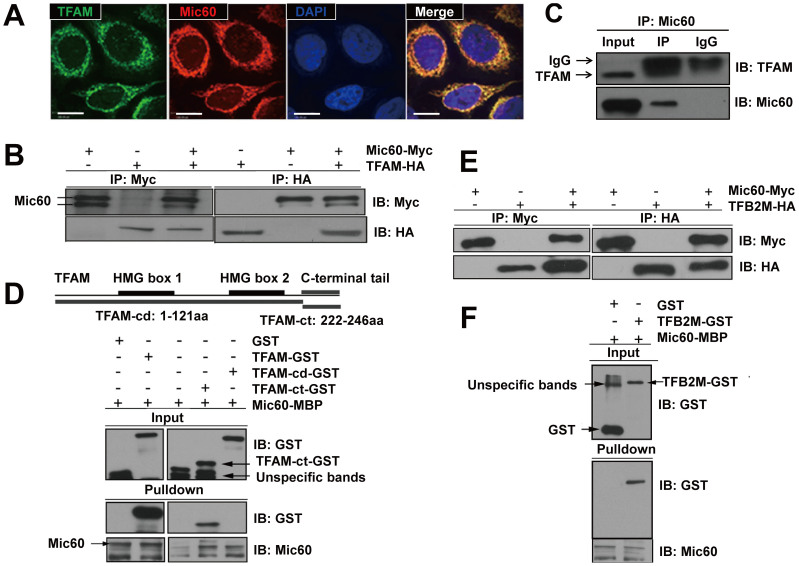
Mic60 interacts with TFAM and TFB2M. (A) Immunostaining of HeLa cells with antibodies against Mic60 and TFAM. HeLa cells were fixed and incubated with the antibodies specific with Mic60 and TFAM. Secondary antibodies labeled with TRITC (for antibody against Mic60, red) and FITC (for antibody against TFAM, green) were incubated further. DAPI was used to stain the nuclear DNA (blue). Scale bars indicate 10 μm. (B) Co-immunoprecipitation (IP) of Mic60 and TFAM in cell lysates using antibodies specific for the c-Myc tag or the HA tag. Cell lysates were obtained from HEK293T cells transfected with Mic60-Myc and TFAM-HA. (C) Co-IP of endogenous Mic60 and TFAM in HEK293T cell lysates. Input, 10%. Immunoblotting assays were performed after IP. (D) *In vitro* binding assays showed that the C-terminal tail of TFAM was indispensable for the interaction (Input, 5%) with Mic60. The functional domains of TFAM are represented on the top row of panel D. TFAM C terminal (ct) and TFAM with a C-terminal tail deletion (TFAM-cd), which was used for the *in vitro* binding assays, are depicted. Mic60 was immobilized on resin and incubated with recombinant TFAM-GST, TFAM-cd-GST or TFAM-ct-GST proteins that were prepared by GST purification. The bound complexes were analyzed by immunoblotting. (E) Co-IP of Mic60 and TFB2M in cell lysates were from HEK293T cells expressing Mic60-myc together with TFB2M-HA. Input, 5%. (F) *In vitro* binding assays for Mic60 and TFB2M. TFB2M-GST purified from bacteria incubated with Mic60-MBP protein and the protein complexes were analyzed by immunoblotting. Input, 10%.

TFAM is a crucial component of the basal mitochondrial transcription machinery and is also involved in the packaging of mitochondrial nucleoids^19^. Through immunofluorescence, we observed that Mic60 and TFAM were partially co-localized in mitochondria (Figure 1A). To examine whether Mic60 interacts with TFAM, we performed co-immunoprecipitation (co-IP) experiments using the lysates isolated from HEK293T cells overexpressing Mic60-Myc and TFAM-HA. Results showed that Mic60 and TFAM co-immunoprecipitated (Figure 1B). To confirm this observation, we performed co-IP of native Mic60 and TFAM in HEK293T cells. The endogenous IP results confirmed the interaction between Mic60 and TFAM (Figure 1C). To map the Mic60-TFAM interaction regions, full-length TFAM and truncated TFAM with a GST tag and Mic60 with an MBP tag were used for *in vitro* binding assays. As shown in Figure 1D, Mic60-MBP bound to a full-length TFAM-GST fusion protein, but not to the GST control. Furthermore, we observed that Mic60 bound to the truncated TFAM-ct (C terminus) but showed no binding to the HMG box domain of TFAM (Figure 1D). These results indicate that Mic60 interacts with TFAM.

The interaction between Mic60 and TFAM prompted us to ask whether Mic60 also interacts with other mitochondrial transcription factors. We further examined the interaction between Mic60 and TFB2M, another component of the basal mitochondrial transcription machinery. Immunoaffinity purification and co-IP experiments showed that Mic60-Myc interacted with TFB2M-HA (Figure 1E), and GST pulldown assays confirmed this interaction (Figure 1F). In addition to the basal mitochondrial transcription factors (TFAM and TFB2M), co-IP results also showed the interaction of Mic60 with a TFB2M paralog, TFB1M (Supplementary figure S1). These results indicate that Mic60 interacts with mitochondrial transcription factors within the transcription machinery and suggest a potential involvement of Mic60 in mtDNA transcription.

### Mic60 knockdown decreases mtDNA transcripts and OXPHOS activities

**Figure 2:**
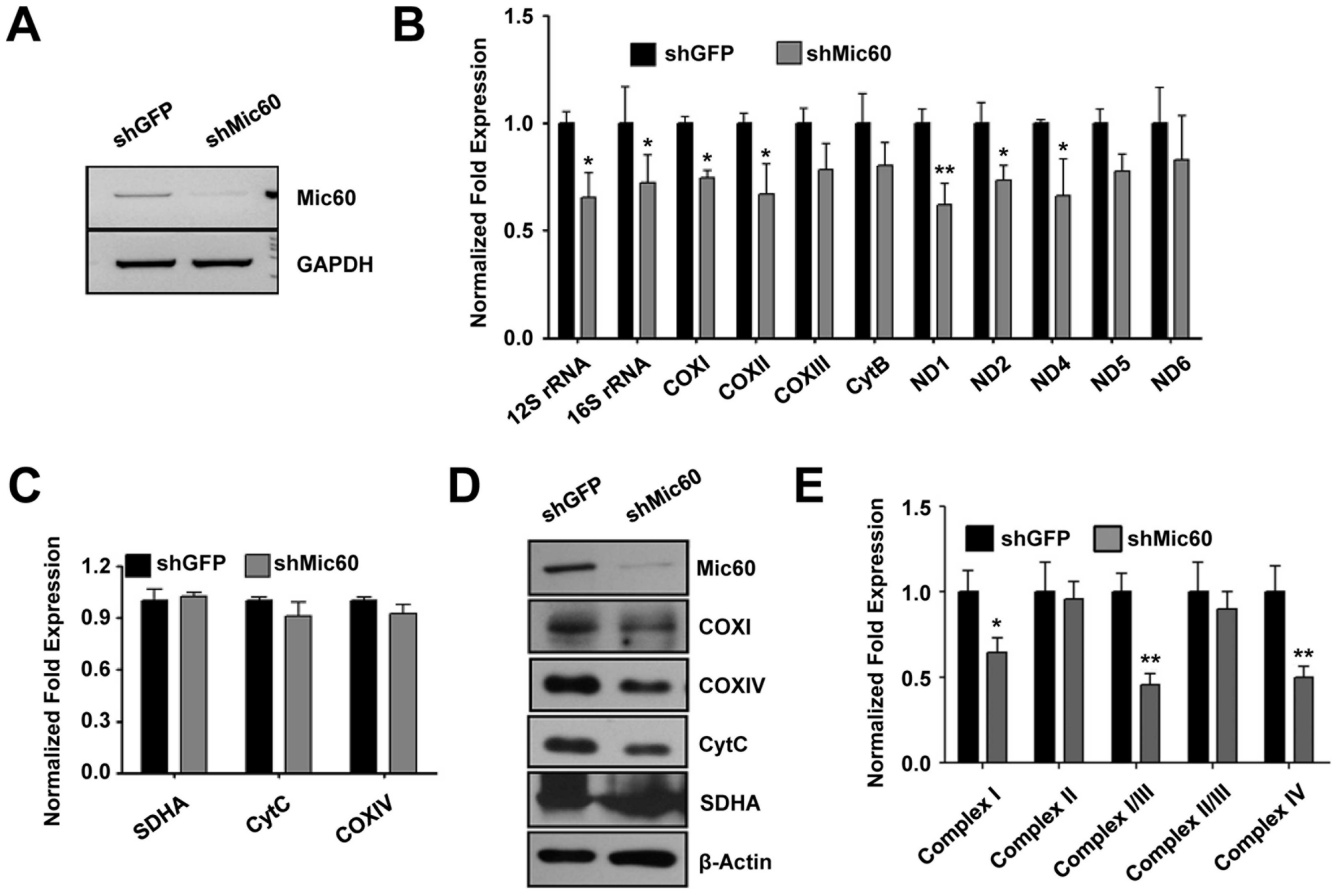
Mic60 knockdown decreases mtDNA transcripts and OXPHOS activities. (A) RNA-mediated knockdown of Mic60 in the HeLa cells. Reverse transcription-PCR analysis of Mic60 expression from HeLa cells infected with pSIREN-RetroQ-shMic60 (shMic60) or pSIREN-RetroQ-shGFP (shGFP)-packaged virus using primers specific for Mic60 and GAPDH. (B and C) Transcription levels of mtDNA- and nuclear DNA-encoded subunits in HeLa cells expressing either a control GFP shRNA or Mic60 shRNA. Real-time PCR analysis of mRNA from HeLa cells expressing either a control GFP shRNA or Mic60 shRNA. Primers used specifically amplify the OXPHOS subunits and GAPDH. The independent experiments were repeated at three times and data shown are means ± SD. (D) Immunoblotting analysis of mtDNA- or nuclear DNA-encoded protein extracts obtained from HeLa cells expressing either a control GFP shRNA or Mic60 shRNA. (E) The mitochondria of Mic60-knockdown and control cells were isolated and used for measuring respiratory chain complex activities. The independent experiments were repeated at three times and data shown are means ± SD. **P < 0.01 or *P < 0.05 for Mic60-knockdown cells versus control cells.

To examine the role of Mic60 in mtDNA transcription, we knocked down Mic60 in HeLa cells using a retrovirus containing Mic60-shRNA (Figure 2A) and examined the steady-state levels of mRNA and rRNA transcribed from mtDNA through real-time PCR. Results showed that, to different extents, the expression levels of mtDNA transcripts, from either the heavy strand or the light strand, were generally decreased in Mic60-knockdown cells compared with those in control cells (Figure 2B). We further analyzed transcripts and protein levels of the nuclear DNA-encoded subunits, including cytochrome c oxidase subunit IV (COXIV), cytochrome c and succinate dehydrogenase complex subunit A (SDHA) in Mic60-knockdown cells. Although the protein levels of COXIV and cytochrome c were reduced upon Mic60-knockdown, all the nuclear DNA-encoded genes showed no significant change in their mRNA levels upon knockdown of Mic60 (Figure 2C and 2D). These results indicate that Mic60 deficiency results in a specific decrease in mtDNA transcription levels.

To investigate further the importance of mtDNA-encoded genes to mitochondrial function, we next analyzed the enzyme activities of OXPHOS complexes. The results showed that the activities of the OXPHOS complexes containing mtDNA-encoded subunits, including complexes I, III, IV, were decreased significantly in Mic60-knockdown cells, whereas the activity of complex II (which is exclusively constituted by nuclear DNA-encoded subunits) in Mic60-knockdown cells was comparable to that of control cells (Figure 2E). These results suggest that Mic60 deficiency compromises OXPHOS activities, and this effect is at least in part caused by the decrease in mtDNA transcription.

### Suppression of Mic60 decreases TFAM binding to mtDNA promoters

**Figure 3:**
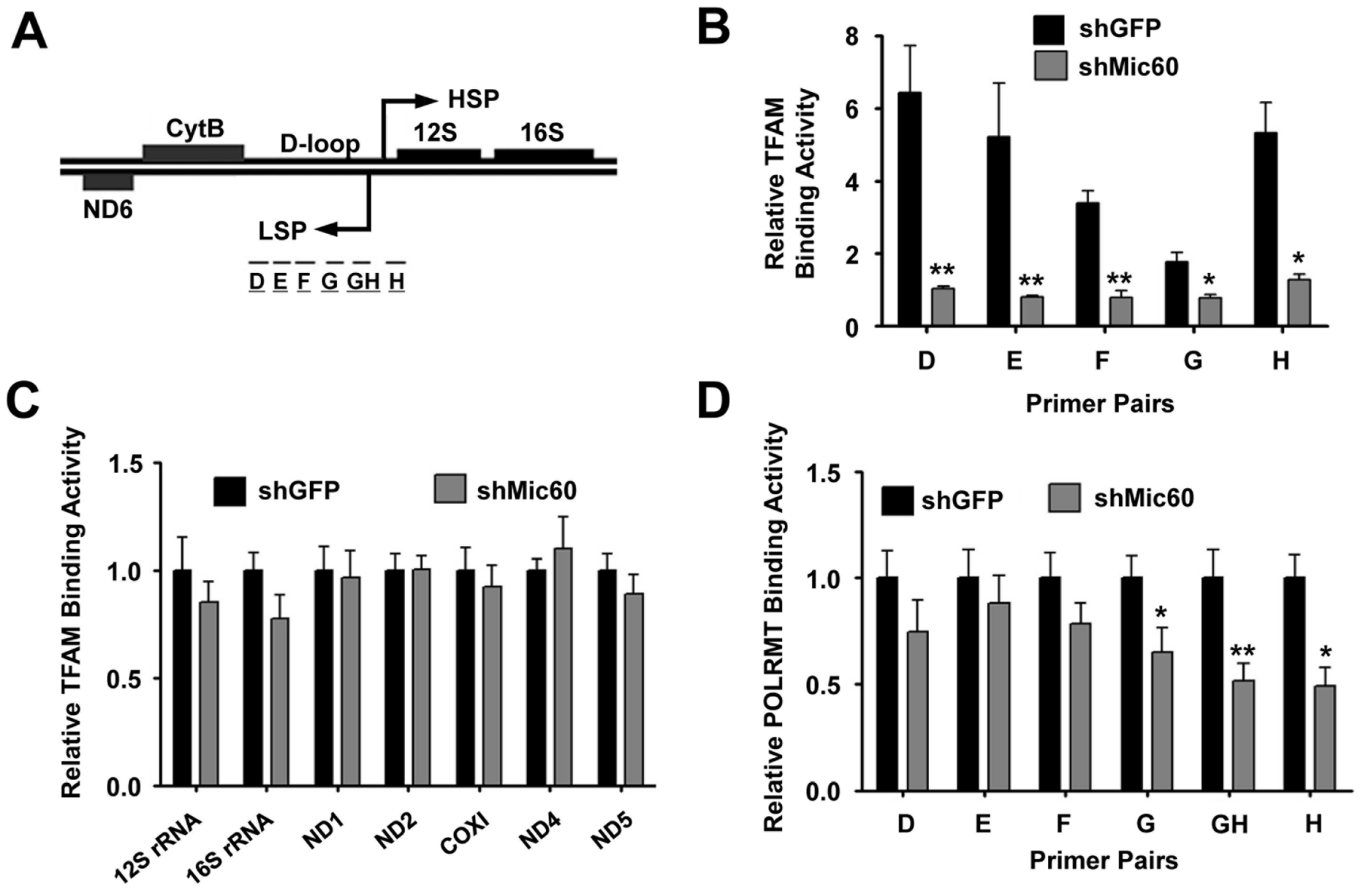
Mic60 deficiency decreases TFAM binding to mtDNA promoters. (A) Schematic representation of the primer pairs used for mtDNA immunoprecipitation (mIP) assays. D-loop regions and adjacent mtDNA-encoded subunits are depicted. The primer pairs were described previously^21^. (B and C) Control (shGFP) and Mic60-knockdown (shMic60) cells were subjected to mIP assay with antibody recognizing TFAM. The association of TFAM with the promoter region and across the genes were analyzed as indicated. Primer pairs used are depicted in panel A. (D) HeLa cells expressing either GFP shRNA (shGFP) or Mic60 shRNA (shMic60) were subjected to mIP assay using antibodies recognizing POLRMT. For (B) to (D), the independent experiments were repeated at three times and data shown are means ± SD. Compared with the shGFP control cells **P < 0.01 or *P < 0.05. HSP, heavy strand promoter; LSP, light strand promoter.

Considering that TFAM binding to the mtDNA promoters is of prime importance for mtDNA transcription^5^,^20^, we next sought to examine whether Mic60 affects mtDNA transcription by changing the concentration of TFAM at mtDNA promoters. To test this possibility, we analyzed TFAM distribution in the D-loop region in control and Mic60-knockdown cells by mtDNA immunoprecipitation (mIP). Five pairs of primers complementary to different parts of the D-loop region (primer sets D to H) were used to examine the extent of binding with TFAM^21^ (Figure 3A). In Mic60-knockdown cells, significantly lower TFAM binding signals were detected throughout the D-loop region (Figure 3B). In contrast, no significant change in TFAM concentration was observed in mtDNA coding regions, including in regions encoding mitochondrial rRNAs, NADH dehydrogenase subunits (NDs) and cytochrome c oxidase subunit I (COXI) (Figure 3C). These results indicate that Mic60 is important for the specific binding of TFAM to mtDNA promoters.

Additionally, considering that mitochondrial RNA polymerase POLRMT must be recruited to TFAM to transcribe mtDNA, we further performed an mIP assay to examine the effect of Mic60 deficiency on POLRMT recruitment. Consistent with the results of TFAM distribution, the POLRMT binding signals at the D-loop region, especially around mtDNA promoters (corresponding to primer sets G to H) were also weaker in Mic60-knockdown cells compared with control cells (Figure 3D). Hence, the suppression of Mic60 decreases TFAM binding and POLRMT recruitment to mtDNA promoters, which may contribute to the decline in mitochondrial transcription.

### Mic60 interacts with mtDNA and is involved in the architecture of the mtDNA D-loop region

**Figure 4:**
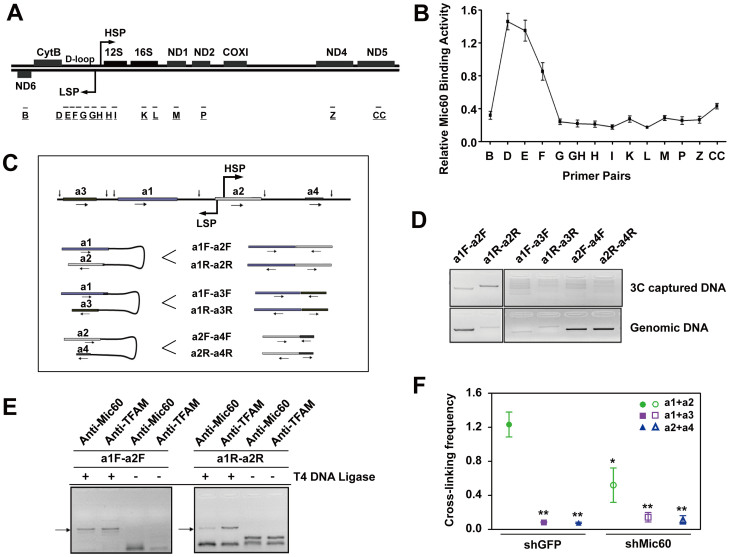
Mic60 contacts with mtDNA and is involved in the D-loop architecture. (A) Schematic representation of the primer pairs used for mIP assays. D-loop regions and several mtDNA-encoded subunits are depicted. The primer pairs were described previously^21^. (B) mIP assays performed in HeLa cells using an antibody specific for Mic60. Mic60 enrichment was shown to locate in the DNA non-coding region and peaked around the D and E fragments. Independent experiments were repeated three times and data are shown are means ± SD. (C) Schematic representation of 3C design and proposed ligation fragments. Ase I restriction endonuclease sites are depicted by arrows above the sequence. The primer sets used in the Ase I-digested samples are depicted, with F representing the forward direction of DNA and R representing the reverse direction of DNA. (D) 3C analysis of Ase I-digested samples. PCR products were obtained from the a1-a2 ligated fragments. (E) ChIP-3C assays showed that Mic60 and TFAM were located in the loop structure of mtDNA regulatory region. Normal rabbit IgG or antibodies specific for Mic60 or TFAM were used. Unligated samples were used as negative controls. Arrows indicate the 3C captured DNA products of expected lengths. (F) Cross-link frequency determination of Ase I-digested samples by 3C assays. Mic60 expression was knocked down in Hela cells using shRNA. Cross-linking frequencies were determined in triplicate, and means and SEM are plotted for every primer pair. Compared with shGFP ‘a1 + a2' results, *P < 0.05, **P < 0.01. HSP, heavy strand promoter; LSP, light strand promoter.

Given that Mic60 interacts with mitochondrial transcription factors and affects TFAM binding to mtDNA promoters, we asked whether Mic60 interacts with mtDNA. Previous studies have shown that mtDNAs are packaged into nucleoids with layered factors^22^,^23^, and most nucleoids contact with mitochondrial inner membrane/cristae^24^. Mic60 is an inner membrane protein that controls cristae morphology^14^, where it could potentially interact with nucleoids. To examine the possibility that Mic60 interacts with mtDNA, we performed mIP assays *in vivo*. Fourteen pairs of primers corresponding to the regulatory D-loop region and its adjacent coding regions were used as previously reported^21^ (Figure 4A). mtDNA fractions enriched with Mic60 was eluted and used for real-time PCR analysis. Distinct and strong signals were detected in the upstream region of the mtDNA heavy strand promoter (using primer sets D to F), whereas the other regions of mtDNA showed only baseline levels of Mic60 interaction (Figure 4B). These mIP results suggest that Mic60 interacts with mtDNA, particularly in the region upstream of the heavy strand promoter.

Previous studies have reported that TFAM is capable of forcing promoter DNA to form a U-turn structure^7^,^8^. Hence, a correct conformation of the mtDNA regulatory region might be important to mtDNA transcription. Since Mic60 interacts with mtDNA and mtDNA transcription factors, we hypothesized that Mic60 might be involved in the formation of higher-order mtDNA architecture. Chromatin conformation capture (3C) assay generates a population-average measurement of the frequency of juxtaposition of any two genomic loci due to interaction with a common protein or proteins, and thus provides information about their relative proximity^25^,^26^. In our previous studies, we employed this technique to investigate higher-order chromatin structures in the regulation of hemoglobin gene expression^25^,^27^. Here, to assess the formation of hypothetical higher-order mtDNA architecture, we applied the 3C methodology to mtDNA. We firstly used the restriction enzyme CviA II for mtDNA 3C assays and designed PCR primer sets flanking the CviA II sites (Supplementary Figure S2A). Consistent with a previous study^28^, a PCR product was highly amplified from the DNA fragment ligating b and c (primer sets bF-cF and bR-cR) indicating that there was a specific loop between the mtDNA promoter and the mtDNA transcription terminator (Supplementary Figure S2B-S2D). In addition, the PCR product was also highly amplified from the DNA fragment ligating a and b (primer sets aF-bF and aR-bR, Supplementary Figure S2B-S2D). In contrast, we did not detect marked PCR products from DNA fragments ligating the adjacent regions a and d, or regions c and e (primer sets aF-dF, aR-dR, cF-eF and cR-eR, Supplementary Figure S2B and S2C). These results indicate that loci within the mtDNA D-loop region associate with each other and form higher-order architecture. To further validate this D-loop region architecture, we used another restriction enzyme, Ase I, in 3C assays. Ase I was used because its recognition sites and those of CviA II have similar locations relative to the mtDNA D-loop region. In agreement with the results using CviA II, the PCR product generated from the Ase I cut mtDNA fragment ligating a1 and a2 (around mtDNA promoters) showed a high cross-linking frequency between these two regions, whereas products generated from other ligated regions (a1 and a3, a2 and a4) were very weak or undetectable (Figure 4C–4D). These results confirm that a higher-order structure is formed within the D-loop region.

To test whether Mic60 is involved in the D-loop region architecture, we performed ChIP-3C assays. Restriction enzyme Ase I was used to digest DNA. After immunoprecipitation using antibodies against Mic60 and TFAM, respectively, ligated fragments were analyzed by PCR. We detected PCR products of the expected sizes using the primer sets a1F-a2F and a1R-a2R in both Mic60's co-precipitates and TFAM's, which means that mtDNA fragment ligating a1 and a2 interacts with Mic60 and TFAM (Figure 4E). These results suggest that Mic60, as well as TFAM, is involved in the architecture of the mtDNA D-loop region. To investigate the importance of Mic60 in the higher-order structure formed within the D-loop region, we knocked down Mic60 and performed 3C assays. Through real-time PCR, a specific high linking frequency was detected between regions a1 and a2 in shGFP control cells. However, in shMic60 cells, the linking frequency between a1 and a2 regions was not significantly different from the a1-a3 and a2-a4 linking frequencies and was markedly lower than that in shGFP control cells (Figure 4F). These results indicate that Mic60 is important for the architecture of mtDNA D-loop region, which may contribute to the importance of Mic60 in mtDNA transcription and respiration.

## Discussion

Mic60 plays an important role in mitochondrial morphology, mitochondrial functions and ROS production^14^,^29^. Although Mic60 is well known for the organization of the mitochondrial inner membrane as a component of the mitochondrial contact site and cristae organizing system (MICOS)^15^,^16^, little is known about other functions of Mic60. In the present study, we found that Mic60 interacted with mitochondrial transcription factors TFAM, TFB2M and TFB1M and that Mic60 deficiency resulted in a decrease in mtDNA transcription. Mechanistically, we demonstrated that suppression of Mic60 decreased TFAM binding and POLRMT recruitment to mtDNA promoters, which may contribute to the downregulation of mtDNA transcription and OXPHOS activities. Additionally, we found that Mic60, interacted with mtDNA and was involved in the architecture of mtDNA D-loop region. Our findings reveal a previously unknown role of Mic60 in mtDNA transcription.

Mic60 was at first identified as a transmembrane protein in mitochondrial inner membrane in the heart and thus is also known as heart muscle protein (HMP)^13^. As an organizer of the MICOS or MINOS (mitochondrial inner membrane organizing system) complex at mitochondrial cristae junctions, Mic60 controls mitochondrial cristae morphology and is also coupled to the outer membrane to promote protein import via the mitochondrial intermembrane space assembly pathway^14^,^30^,^31^,^32^,^33^. Mic60 knockdown results in both structural and functional changes with increased ROS production and membrane potential^14^. Very interestingly, our previous study showed that the overexpression of Mic60 in cardiac muscle cells also increased ROS production and aggravated cardiac hypertrophy^18^. These seemingly paradoxical phenomena suggest a tightly controlled amount of Mic60 for cellular homeostasis. In addition, our previous work showed that Mic60 also regulates apoptosis by controlling cytochrome c release from the intermembrane to the cytosol^17^. While in the present study, we found that Mic60 interacts with the mtDNA transcription machinery and is important for a normal function of OXPHOS. This finding unveils a so far unrecognized aspect of Mic60's function in mitochondrial biology.

Recent studies demonstrated that membrane proteins of mitochondria are involved in mtDNA stability and nucleoids packaging^17^,^34^,^35^,^36^,^37^,^38^. Most mitochondrial nucleoids are supposed to be anchored in the inner membrane^39^,^40^. In a previous report, Mic60 was identified in nucleoids through mass spectrometry^22^. Consistently, we observed that Mic60 could interact with mtDNA through mIP. Moreover, our results showed that TFAM occupancy in the mtDNA promoters was reduced, and mtDNA transcripts were decreased upon Mic60-knockdown. These results suggest that Mic60 may act as an organizer providing the optimal structure for mtDNA transcription in the nucleoids. Although we could not completely exclude the possibility that the compromised mitochondrial transcription and OXPHOS activities were secondary to morphological defects caused by Mic60 deficiency, our findings suggest that the decrease in mtDNA transcription is at least partially caused by lower TFAM binding and POLRMT recruitment to mtDNA promoters upon Mic60 knockdown.

TFAM, TFB2M and POLRMT constitute the basal mtDNA transcription machinery that is sufficient to initiate mtDNA transcription *in vitro*^5^. TFAM functions as an activator of mtDNA transcription and packages mtDNA into a DNA-protein macrocomplex^11^,^41^,^42^. TFAM also binds to, bends and shapes mtDNA into a sharp U-turn DNA structure^7^,^8^. In the present study, we demonstrated that Mic60 interacts with core mitochondrial transcription factors, including TFAM and TFB2M. Mic60 interacts with the C-terminus of TFAM which is vital for mtDNA transcription, and Mic60 knockdown decreases TFAM binding to mtDNA promoters. Hence, Mic60 is important for TFAM dependent mitochondrial transcription initiation. Additionally, we found that both Mic60 and TFAM are involved in the higher-order structure of the mtDNA D-loop region. Currently, we do not know in detail how this higher-order structure is built with Mic60 and additional factors, but we found that Mic60 is important for the higher-order structure of the D-loop region and we speculate that Mic60 might help to keep TFAM binding to the promoters within this D-loop architecture, which guarantees a high transcriptional efficiency.

In the present study, we found that, in addition to the well-characterized MICOS subunits, Mic60 interacts with mitochondrial transcription factors. Suppression of Mic60 compromises mitochondrial transcription and OXPHOS function. Moreover, we found that Mic60, as well as TFAM, is involved in the higher-order D-loop region architecture. Mic60 is important for TFAM binding and POLRMT recruitment to mtDNA promoters. Our findings reveal a new function of Mic60 outside cristae junction organization, and shed new light on the intimate interactions between membrane proteins and nucleoids in mitochondria.

## Methods

### Cell culture

HEK293T and HeLa cells were cultured in an atmosphere of 5% CO_2_ at 37°C in Dulbecco's modified Eagle's medium (DMEM, Invitrogen) supplemented with 10% fetal bovine serum (Invitrogen).

### Plasmids and subcloning

Wild-type forms of human Mic60, TFAM and TFB2M, each tagged at its C-terminus with c-Myc or HA epitopes, were subcloned into pcDNA4 (Invitrogen). Wild-type or mutant forms of human Mic60 were subcloned into pET-42a (Novagen) and pMAL-4X (New England Biolabs). Wild-type and mutant human TFAM and TFB2M were tagged with an HA epitope at their C-terminus and subcloned into pET-42a.

### Antibodies and immunoblotting

Proteins (30 μg) were separated by SDS-PAGE and transferred onto polyvinylidene difluoride (PVDF) membranes (Millipore). Membranes were probed with specific antibodies; blots were washed and probed with corresponding peroxidase-conjugated secondary antibodies. The following antibodies were used: rabbit polyclonal antibody specific for Mic60 (Novus; ABcam), rabbit polyclonal antibody specific for COXIV (Cell Signaling Technology), goat polyclonal antibody specific for TFAM (Santa Cruz Biotechnology), rabbit polyclonal antibody specific for cytochrome c (Santa Cruz Biotechnology), mouse monoclonal antibody specific for c-Myc (Santa Cruz Biotechnology), mouse monoclonal antibodies specific for COXI and SDH (Mitosciences), and mouse monoclonal antibodies specific for HA and beta-actin (Sigma-Aldrich). Primary antibodies were used at a 1:1,000 dilution, and secondary antibodies were used at a 1:5,000 dilution for immunoblotting.

### Immunofluorescence staining

HeLa cells were seeded on glass coverslips placed in 24-well plates. Cells were fixed with freshly prepared 4% paraformaldehyde for 15 min, followed by permeabilization with 0.2% Triton X-100 in PBS for 5 min. Next, the slides were washed in PBS containing 10% goat serum and incubated overnight with primary antibodies at 4°C. We used antibodies against Mic60 and TFAM as described above. The slides were then washed with PBS and incubated with the appropriate secondary antibodies labeled with TRITC (for antibody against Mic60) or FITC (for antibody against TFAM) (Invitrogen) for 1 hour at 37°C. The nuclei were stained with DAPI. The images were obtained using a fluorescence microscope (OLYMPUS DX51, Japan) and DP2-BSW software (version 2.2).

### Immunoprecipitation

Whole-cell extracts were prepared using extraction buffer (10 mM Tris-HCl at pH 7.5, 150 mM NaCl, 1 mM EDTA, 0.5% NP-40 and 1 mM DTT) supplemented with a protease inhibitor cocktail (Sigma-Aldrich). For immunoprecipitations, equal amounts of lysate (containing 1–2 mg of total cellular protein) were pre-cleared with rabbit IgG or mouse IgG (Santa Cruz Biotechnology) and protein A/G plus beads (Millipore). Pre-cleared extracts were incubated with Protein A/G plus beads (Millipore) and 2 μg rabbit polyclonal antibody specific for Mic60 or mouse monoclonal antibodies specific for c-Myc or HA. Precipitates were washed extensively in extraction buffer, and bound complexes were eluted with 2× SDS-PAGE sample buffer and analyzed by immunoblotting.

### Protein purification and *in vitro* binding assays

GST fusion proteins of TFAM, C-terminal tail of TFAM (TFAM-ct), C-terminal tail deletion (TFAM-cd), Mic60 and TFB2M were expressed in *E. coli* and purified from the IPTG (isopropyl-β-D-thiogalactopyranoside)-induced bacterial pellet by sonication in a buffer (Tris-HCl at pH 8.0, 100 mM NaCl, 0.1 mM EDTA, 1% Triton X-100, 1 mM DTT and 1 mM PMSF). The supernatant was applied to a glutathione-sepharose 4B column (GE). The columns were washed, and the proteins were eluted with glutathione (5 mM) in the same buffer and then dialyzed against a Tris-HCl buffer (10 mM at pH 7.9) containing NaCl (100 mM). Control GST protein was purified as described above. The MBP fusion proteins of Mic60, M1, M2 and M3, were expressed and sonicated under the conditions as described above. The supernatants were applied to a resin and incubated with the purified TFAM, TFAM-ct, TFAM-cd, and TFB2M GST fusion proteins. Bound complexes were washed, eluted and analyzed by immunoblotting.

### RNAi

Cell lines stably expressing GFP-shRNA and Mic60-shRNA were established using a vector-based shRNA technique. shRNA was designed by Invitrogen and subcloned into pSIREN-RetroQ. The Mic60 shRNA sequence was reported previously^17^. Retroviruses were produced by co-transfecting subconfluent HEK293T cells with one of the expression plasmids and packaging plasmids (pMD and pVSVG) using Lipofectamine 2000 (Invitrogen) as a transfection reagent. Infectious retroviruses were collected 48 h after transfection, centrifuged to remove cell debris and filtered through 0.45 μm filters (Millipore). HeLa cells were transduced with the retrovirus containing Mic60 or GFP shRNA. Mic60 knockdown efficiency was determined by real-time PCR and immunoblotting.

### RNA isolation, reverse transcription (RT) and real-time PCR

Total RNA was extracted from HeLa cells using Trizol total RNA isolation reagent (Invitrogen) according to the manufacturer's instructions and then treated with DNase I. cDNA was synthesized from total RNA. To determine the levels of nuclear DNA- and mtDNA-encoded mitochondrial gene expression, primers were designed using Primer Premier 5 software, and real-time PCR was performed using SYBR Green PCR Mix (Takara) with the Applied Biosystems 7500 and Bio Rad IQ5 real-time PCR systems. Relative expression levels were calculated from a relative standard curve obtained using log dilutions of cDNA containing the gene of interest. The mean of three independent values for each gene and sample was calculated and normalized to the endogenous reference control gene GAPDH. PCR primer pairs were provided in Supplementary Table S1.

### Mitochondrial DNA immunoprecipitation (mIP)

HeLa cells were fixed in 1% formaldehyde at 37°C for 10 min. The crosslinking reaction was quenched by adding 125 mM glycine, and the cells were incubated for an additional 5 min. Cells were washed twice with ice-cold phosphate-buffered saline, scraped, and centrifuged at 4°C. Cell pellets were resuspended in lysis buffer and sonicated to shear the DNA. After sonication, the lysate was centrifuged, and the supernatant was diluted 5-fold with IP buffer (0.01% SDS, 1% Triton X-100, 2 mM EDTA, 10 mM Tris-HCl pH 8.1, 150 mM NaCl and protease inhibitors). Rabbit antibody specific for Mic60, goat antibody specific for TFAM, normal rabbit IgG or normal goat IgG was added to the supernatant and incubated at 4°C overnight with rotation. The immunocomplexes were precipitated with protein A/G-agarose and sequentially washed with low-salt buffer, high-salt buffer and lithium chloride wash buffer and eluted with elution buffer (1% SDS, 0.1 M NaHCO_3_). Reversal of crosslinking was performed by heating samples in the presence of NaCl and proteinase K at 65°C overnight. DNA was extracted once with phenol/chloroform (1:1) and once with chloroform and precipitated with 2.5 volumes of ethanol in the presence of 3 M NaAc (pH 5.2) at −20°C overnight. The amount of immunoprecipitated DNA was analyzed by real-time PCR using Bio-Rad IQ5 system with SYBR green PCR mix. Real-time PCR Primers for the ‘GH' region are TATTGATGAGATTAGTAGTATGGGAGTG (forward) and CCAAAAACAAAGAACCCTAACAC (reverse). Other primers were described previously^21^.

### 3C and ChIP-3C

We fixed and extracted DNA-protein complexes from HeLa cells as described for the mIP protocol. To wash out any non-cross linked proteins from the mtDNA, SDS was added to a final concentration of 0.1%, and the reaction mixture was incubated at 37°C for 10 min. Triton X-100 was then added to a final concentration of 1% to sequester the SDS and allow the subsequent restriction enzyme digestion. The mtDNA was digested with a high concentration of the enzyme CviA II and Ase I (New England Biolabs) at 37°C for 12 h. After heat inactivation of the restriction enzymes, the reaction mixtures were diluted threefold and DNA fragments were ligated using T4 DNA ligase at 16°C for 14 h. DNA was recovered from the ligation mixtures by proteinase K digestion followed by phenol-chloroform extraction and ethanol precipitation and used as a template for PCR amplification with different sets of primers. All ligation products were confirmed by sequencing the respective PCR amplification products. Cross-link frequency was determined as described previously^26^.

For ChIP-3C, DNA-protein complexes were extracted and digested under the same condition as described above. The DNA-protein complexes were immunoprecipitated with antibodies against Mic60 and TFAM, followed by overnight ligation and reverse crosslinking with 4 M NaCl at 65°C for 4 h. For proteinase K digestion, DNA was extracted with phenol-chloroform and precipitated with ethanol. PCR was performed with one tenth of the DNA from each pool. PCR Primers were as described in Supplementary Table S2.

### Statistical analysis

Data were expressed as means ± SD; cross-linking frequencies were shown as means ± SEM. For two groups designed experiments, comparisons were determined using unpaired Student's t-test. Statistical analysis was performed in the SPSS 16.0 statistical program (SPSS, Chicago, IL, USA). P < 0.05 was considered to be statistically significant in the compared groups.

## Author Contributions

D.P.L. obtained the funding and initiated the experiments. D.P.L., R.F.Y., L.H.S. conceived and designed the experiments. R.F.Y., L.H.S., R.Z., Y.Z., Y.X.L., W.Z., Z.Q.Z. and H.Z.C. performed the experiments and analyzed the data. R.F.Y., R.Z., H.Z.C. and D.P.L. wrote the manuscript and all authors reviewed the manuscript.

## Supplementary Material

Supplementary Information Supplementary information
