# Supplementary material for: Suppression of Mic60 compromises mitochondrial transcription and oxidative phosphorylation
Source: Sci Rep. 2015 Jan 23;5:7990. doi: 10.1038/srep07990 (PMC4303897; doi:10.1038/srep07990)
Supplement: Supplementary Information [file srep07990-s1.pdf]

Supplementary information for

# Suppression of Mic60 compromises mitochondrial transcription and oxidative phosphorylation

Rui-Feng Yang[1#], Li-Hong Sun[1#], Ran Zhang[1#], Yuan Zhang[1], Yu-Xuan Luo[1], Wei Zheng[1], Zhu-Qin Zhang[1], Hou-Zao Chen[1] & De-Pei Liu[1]*

1 State Key Laboratory of Medical Molecular Biology, Department of Biochemistry and Molecular Biology, Institute of Basic Medical Sciences, Chinese Academy of Medical Sciences & Peking Union Medical College, Beijing 100005, P.R. China

* Correspondence and requests for materials should be addressed to D.P.L. (liudp@pumc.edu.cn)

[#] These authors contributed equally to this work

**Supplementary Figure S1.**

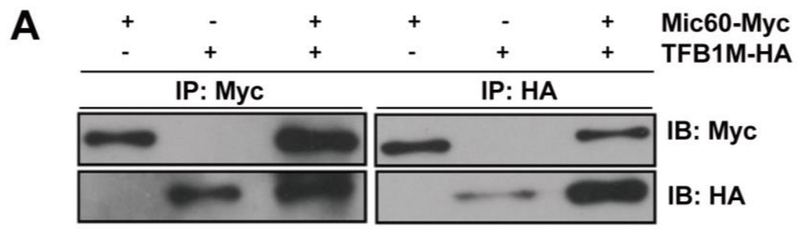


**Supplementary Figure S1. Mic60 interacts with TFB1M.** (A) Co-immunoprecipitation (IP) of Mic60 and TFB1M in cell lysates using antibodies specific for the c-Myc tag or the HA tag. Cell lysates were obtained from HEK293T cells transfected with Mic60-Myc and TFB1M-HA. Input, 10%. Immunoblotting assays were performed after IP.

**Supplementary Figure S2.**

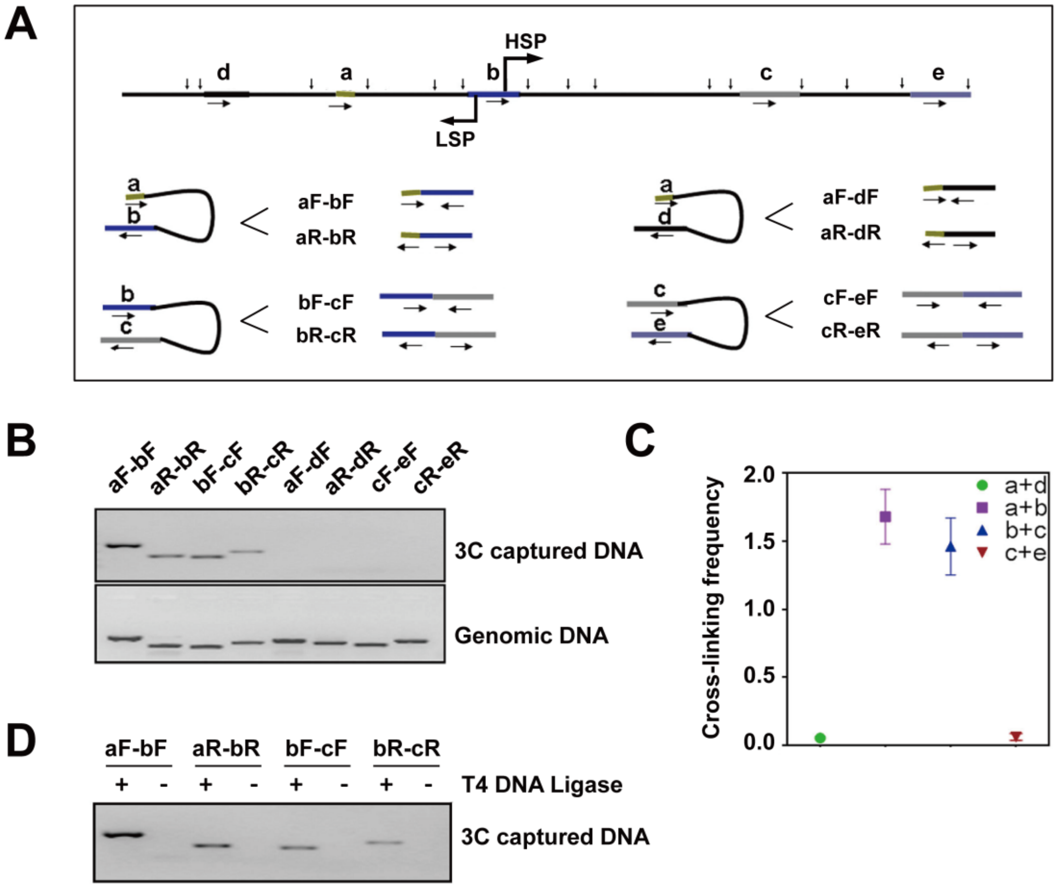


**Supplementary Figure S2. A higher-order mtDNA structure detected by 3C assay using *CviA* II digested fragments.** (A) Schematic representation of 3C design and proposed ligation fragment. *CviA* II restriction endonuclease sites are depicted by arrows above the sequence. The primer sets used in the *CviA* II-digested samples are shown, with F representing the forward direction of DNA and R representing the reverse direction. DNA was digested and intermolecularly ligated for use as a positive control. (B) 3C analysis of *CviA* II-digested samples. As described in Methods, mtDNA-protein complexes were digested by *CviA* II and ligated, and the ligated DNA were eluted for PCR analysis. PCR products were generated with primer pairs aF-bF, aR-bR, bF-cF and bR-cR. (C) Cross-linking frequency determination of *CviA* II-digested samples by 3C assays. Cross-linking frequencies were determined in triplicate, and means and SEM are plotted for every primer pair. (D) 3C analysis of *CviA* II-digested samples. PCR products were obtained between a-b and b-c ligated fragments in the presence of DNA ligase. Unligated samples were used as negative controls.

**Supplementary Table S1.** RT-PCR primers for real-time PCR.

| Gene | Species | Forward primer sequence | Reverse primer sequence |
|---|---|---|---|
| COXI | Mouse | TTGGAGGCTTTGGAAACTGAC | GAGAAGGAGAAATGATGGTGGTAG |
| COXII | Mouse | TGCTCTGAAATTTGTGGATCTAACC | TTTTTTTTTTTTTTTTTTTTTTAAATTAA A |
| COXIII | Mouse | GAAACCACATAAATCAAGCCCTAC | TGAAGAATGTAGAACCATAGATACC |
| Cyto b | Mouse | CCAAATCTCCACGGTCTGTTC | CTCTCCCCAGGTGATGCCT |
| ND1 | Mouse | AGTTCCCCTACCAATACCACACC | GGAGTTTGAGGCTCATCCTGATC |
| ND2 | Mouse | CATAGGGGCATGAGGAGGACT | TGAGTAGAGTGAGGGATGGGTTG |
| ND4 | Mouse | CCTCACATCATCACTCCTATTCTG | GGCTATAAGTGGGAAGACCATTTG |
| ND5 | Mouse | GCCTGATAATAGTGACGCTAGGA | CTATGAATGATTGAGCCAGAGCAT |
| COXIV | Human | CATGTGGCAGAAGCACTATGTGT | GGTTCACCTTCATGTCCAGCA |
| Cyto c | Human | GTGCCAGCGACTAAAAAGAGAAT | AGTCTTGTGCTTGCCTCCCTT |
| SDH | Human | CTGGAGATCCGAGAAGGAAGAG | AGCGAAGATCATGGCTGTCTC |
| 12S rRNA | Human | CACTACGAGCCACAGCTTAA | TCAGGGTTTGCTGAAGATGG |
| 16S rRNA | Human | GGCATGCTCATAAGGAAAGG | GGCCGTTAAACATGTGTCAC |
| COXI | Human | GATTTTTCGGTCACCCTGAAG | CTCAGACCATACCTATGTATC |
| COXII | Human | CTATCCTGCCCGCCATCATC | GATTAGTCCGCCGTAGTCGG |
| COXIII | Human | CACATCCGTATTACTCGCATC | GAAGTACTCTGAGGCTTGTAG |
| Cyto b | Human | CAAACTAGGAGGCGTCCTTG | CTGGTTGTCCTCCGATTCAG |
| ND1 | Human | CCTAGGCCTCCTATTTATTC | GAATGATGGCTAGGGTGAC |
| ND2 | Human | CTACGCCTAATCTACTCCAC | CTTTGAAGGCTCTTGGTCTG |
| ND4 | Human | GGACTCCACTTATGACTCCC | GGTTGAGAATGAGTGTGAGGC |
| ND5 | Human | CTATCACCACTCTGTTCGCAG | GTGGTTGGTTGATGCCGATTG |
| ND6 | Human | CTAAAACACTCACCAAGACC | GGAATGATGGTTGTCTTTGG |
| Mitofilin | Human | GGGTACAAGAACAGGAATTGAAGT | AGAAACGGGCTCTAAGGGTCTC |
| GAPDH | Human | TGTGGGCATCAATGGATTTGG | ACACCATGTATTCCGGGTCAAT |

**Supplementary Table S2.** PCR primers for 3C assays.

| Primer | Primer sequences |
|---|---|
| aF | TGTGGGGGGTGTCTTTGG |
| aR | CCACAGCACTTAAACACATCTCTG |
| bF | GGGAGGGGGTTTTGATGTG |
| bR | GTACCATAAATACTTGACCACCTGTAG |
| cF | GCGTGAAGGTAGCGGATGA |
| cR | ACTTTATTGACTCCTAGCCGCAG |
| dF | AACTCACTGGAACGGGGATG |
| dR | GACCTGGCGGTGCTTCATATC |
| eF | GCGAGAATAATGATGTATGCTTTG |
| eR | AACAGAAACAAAGCATACATCATTATTC |
| a1F | AAAAAAGTAAAAGGAACTCGGCAAAC |
| a1R | CGGAGATGTTGGATGGGGTG |
| a2F | GGATAATGCCGATGTTTCAGGTT |
| a2R | ACACAGCAAGACGAGAAGACCCT |
| a3F | GCCAAGGAGTGAGCCGAAGTT |
| a3R | AGCCTGCGTCAGATCAAAACACT |
| a4F | CCTACAAACAACTAACCTGCCACT |
| a4R | TCTTCTATGATAGGGGAAGTAGCGT |
